# StructureMan: A Structure Manipulation Tool to Study Large Scale Biomolecular Interactions

**DOI:** 10.3389/fmolb.2020.627087

**Published:** 2021-01-11

**Authors:** Yuejiao Xian, Yixin Xie, Sebastian Miki Silva, Chitra B. Karki, Weihong Qiu, Lin Li

**Affiliations:** ^1^Department of Chemistry and Biochemistry, University of Texas at El Paso, El Paso, TX, United States; ^2^Computational Science Program, University of Texas at El Paso, El Paso, TX, United States; ^3^Department of Physics, University of Texas at El Paso, El Paso, TX, United States; ^4^Department of Physics, Oregon State University, Corvallis, OR, United States; ^5^Department of Biochemistry & Biophysics, Oregon State University, Corvallis, OR, United States

**Keywords:** protein-protein interactions, protein-RNA/DNA interactions, electrostatic force, viral capsid assembly, molecular motor, kinesin, DelPhi, DelPhiForce

## Abstract

Studying biomolecular interactions is a crucial but challenging task. Due to their large scales, many biomolecular interactions are difficult to be simulated via all atom models. An effective approach to investigate the biomolecular interactions is highly demanded in many areas. Here we introduce a Structure Manipulation (StructureMan) program to operate the structures when studying the large-scale biomolecular interactions. This novel StructureMan tool provides comprehensive operations which can be utilized to study the interactions in various large biological systems. Combining with electrostatic calculation programs such as DelPhi and DelPhiForce, StructureMan was implemented to reveal the detailed electrostatic features in two large biological examples, the viral capsid and molecular motor-microtubule complexes. Applications on these two examples revealed interesting binding mechanisms in the viral capsid and molecular motor. Such applications demonstrated that the StructureMan can be widely used when studying the biomolecular interactions in large scale biological problems. This novel tool provides an alternative approach to efficiently study the biomolecular interactions, especially for large scale biology systems. The StructureMan tool is available at our website: http://compbio.utep.edu/static/downloads/script-for-munipulation2.zip.

## Introduction

Studying interactions between biomolecules is an important but challenging task. In recent decades, many efforts and progresses have been made to study the biomolecule interactions (Jones and Thornton, 1996; von Mering et al., 2002; Li et al., 2015; Zhou, 2015). Such studies are in two categories: Predicting biomolecule complex structures (Pagadala et al., 2017); and revealing the biomolecule interaction mechanisms (Jones and Thornton, 1996).

To predict the complex structures of biomolecules such as proteins, RNAs/DNAs, many algorithms have been developed based on some physics principals and statistic functions. Some of them are protein-protein docking algorithms (Gabb et al., 1997; Chen et al., 2003; Dominguez et al., 2003; Li et al., 2011), protein-DNA/RNA docking algorithms (Tuszynska and Bujnicki, 2011; Huang et al., 2013; Yan et al., 2017), scoring functions (Chen and Weng, 2003; Jain, 2006; Huang et al., 2010; Li et al., 2013a), etc. To reveal the mechanisms of biomolecular interactions, numerous methods have been developed to simulate the biomolecular binding processes. The two most challenging issues in studying the biomolecular interactions are that the size scale of the biomolecules and time scale of the binding processes. Traditional all atom molecular dynamic simulations can hardly simulate the binding processes of large biomolecular systems, such as capsid proteins binding to a viral capsid. In order to accelerate the large-scale biomolecule simulations, many successful coarse-grained models have been developed (Liwo et al., 1997; Marrink et al., 2007). Such coarse-grained models are in several categories: elastic network models, Go-like models, beads-based models (Tozzini, 2005). Besides coarse-grained models, some multiscale methods have also been developed (Wang et al., 2014; Li et al., 2016b). We have developed a DelPhiForce steered Molecular Dynamic (DFMD) method (Li et al., 2017a; Peng et al., 2019) to speed up the Molecular Dynamic (MD) simulation. The advantage of DFMD is utilizing the long-range electrostatic interactions in the MD simulations to accelerate the binding process. This DFMD method has been proven very successful in protein-biomolecule binding processes. Therefore, studying electrostatic interactions is crucial to investigate large scale biomolecular interactions.

To study the interactions between two biomolecules in various perspectives, the ligand structure needs to be manipulated with respect to the receptor, such as shifted, spun, rotated around the receptor. For some large biomolecules such as viruses, many proteins are required to assembled a complete viral capsid. Studying such large complex structures need more comprehensive manipulations on individual biomolecules. Therefore, we developed a Structure Manipulation (StructureMan) program to manipulate the biomolecule structures. Four basic and two advanced structural operations were developed to manipulate the structures of biomolecules. These basic operations are developed for two biomolecules (a receptor and a ligand), which include separation, spin, rotation and perpendicular shifting between a pair of receptor and ligand. Furthermore, two advanced operations were developed to study the assembly of multiple biomolecules in pseudo spherical or pseudo cylindrical symmetry. The pseudo spherical operations, including capsid generation, capsid expansion and capsid detachment tools, can be widely used to study the viral capsid assembly problems. With StructureMan, users can easily manipulate the structures of a complex to study the electrostatic interactions for large systems, such as protein-protein interactions in a whole virus capsid or a large piece of microtubule. In this work, we applied the StructureMan on a viral capsid and a molecular motor, which demonstrated that this novel tool is very useful when studying large scale biomolecular interactions.

**Figure 1:**
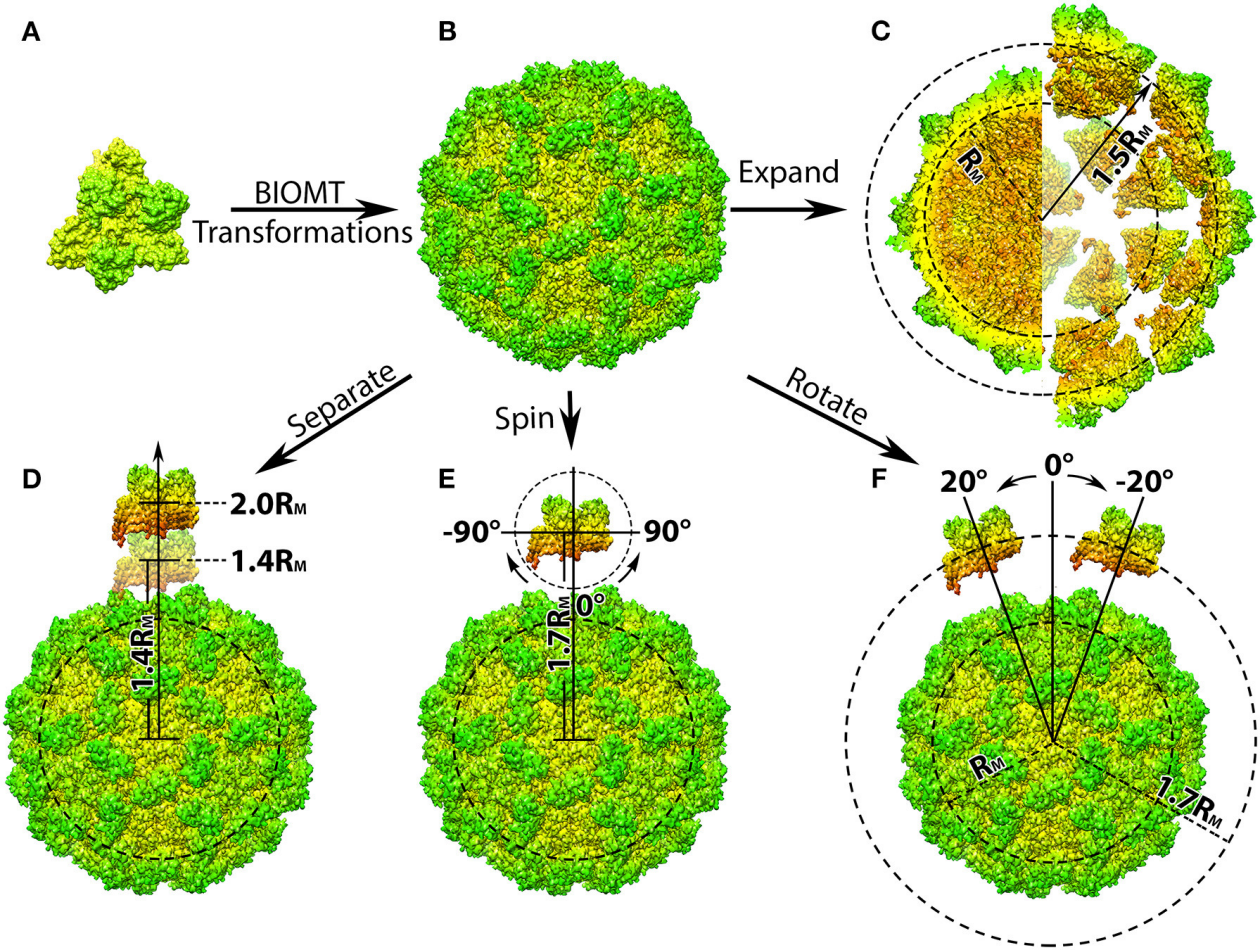
Structure manipulation of the capsid of Turnip Crinkle Virus (TCV). **(A)** Individual capsomer of TCV with the three type of protein subunits labeled on the side (PDB ID 3ZX8); **(B)** the whole TCV capsid generated using the capsid generation tool; Penal **(C–F)** are demonstration of structure manipulation where: **(C)** the native TCV capsid is expended by 0.7R_M_. The central section of the native capsid (left) and the expended capsid (right) are shown as their radius labeled in black, respectively; **(D)** one capsomer is detached from the rest of the capsid by 0.4 to 1.0R_M_, as its distance to the mass center of the whole capsid increases to 1.4R_M_ and 2.0R_M_; **(E)** one capsomer is first detached by 0.7R_M_ and then spun from −90Å to 90Å in the xy-plane (around z-axis); **(F)** after detached by 0.7R_M_, the capsomer is rotated around the capsid from −20Å to 20Å with respect to the mass center of the whole capsid. In all panels, the capsomers and capsids are shown in their density map generated using Chimera (Pettersen et al., 2004; Goddard et al., 2007) and colored by radius from red to green.

Turnip crinkle virus (TCV) is a plant pathogenic virus which is composed of ~4.0 kb plus-sense RNA and 180 copies of capsid protein subunits (Hogle et al., 1986; Wei et al., 1990). These capsid proteins assemble into an icosahedral capsid with a diameter of ~330 Å. For the purpose of this work, the quasi-three-fold symmetry related subunits are grouped together and referred as one capsomer (Figure 1A). The viral capsid has been shown to have multiple functions in stabilizing the genomic RNA materials during viral assembly and protecting RNA and host-defense machinery (Cao et al., 2010). In 2012, an expanded form of TCV was captured and considered to be a putative RNA uncoating intermediate (Bakker et al., 2012). The expanded capsid is resulted from the separation of the capsid proteins. Having its multiple functions and dynamic nature, the TCV capsid is an interesting target for protein-protein interaction studies, especially in the studies of capsid assembly and viral infection (Sorger et al., 1986; Wei et al., 1990; Saunders and Lomonossoff, 2015). As suggested by transmission electron microscopy, the assembly of the TCV capsid is a progressive process where the capsid protein units continuously assemble onto the initiating structure until the viral capsid is completed (Sorger et al., 1986). This assembly process is guided by interactions among the capsid proteins as well as their RNA genome (Sorger et al., 1986; Wei et al., 1990; Bakker et al., 2012; Saunders and Lomonossoff, 2015). In a recent studies, the wild type capsid proteins of TCV expressed in Cowpea Mosaic Virus-Hyper Translatable Expression system self-assembled into TCV-like particles (Saunders and Lomonossoff, 2015). These results suggested that the ability of these capsid protein assemble into the viral capsid is fundamentally essential in TCV's life cycle. Therefore, this study implemented the StructureMan tool to manipulate the structure of TCV capsid, which then facilitated our analyses that reveal the binding mechanisms among capsomers in the TCV capsid. Many interesting features are discovered and shown in the results and discussion section.

Kinesins are a superfamily of molecular motors. Kinesins have vital cellular functions (Mandelkow and Mandelkow, 2002; Endow et al., 2010; Lee et al., 2015; Tseng et al., 2018) in mitosis and become ideal anti-mitotic drug targets for cancer treatment (DeBonis et al., 2004; Tao et al., 2005; Nakai et al., 2009). Traditional anti-mitotic drugs face two significant problems: (1) serious side effects (Jordan and Wilson, 2004; Schmidt and Bastians, 2007); (2) Strong drug resistance for some types of cancers (Kavallaris, 2010). Recent works found that another promising direction of cancer drug design is targeting kinesins (Jackson et al., 2007; Sarli and Giannis, 2008; Huszar et al., 2009). Interrupting the binding or motility of specific kinesins can block the mitosis and kill the cancer cells. Due to the variety types of kinesins (Vale et al., 1985), kinesin targeting drugs will be more selective and also alternative to solve the drugs resistance compared to microtubule targeting drugs. Therefore, discovering and designing drugs targeting certain types of kinesins become a very promising direction for cancer treatment. Efficient drug design approaches highly demand the systematic understanding of binding and motility mechanisms of kinesins. Therefore, many computational studies have been conducted to study the molecular motors including kinesins (Li et al., 2016a,b; Li et al., 2017; Tajielyato et al., 2018). This work utilized StructureMan to study the binding mechanisms between kinesin and microtubule, which sheds light on the drug design targeting the kinesins.

## Methods

Four basic and two advanced structural operations were developed to manipulate the structures of biomolecules. The basic operations are developed for two biomolecules (a receptor and a ligand), which include separation, spin, rotation and perpendicular shifting between a pair of receptor and ligand. Furthermore, two advanced operations were developed to study the assembly of multiple biomolecules in pseudo spherical or pseudo cylindrical symmetry. These pseudo spherical operations, including capsid generation, capsid expansion, and capsid detachment tools, can be widely used to study the viral capsid assembly problems. For the purpose of demonstration of the advanced operations, the protein capsid of the TCV, and the kinesin-microtubule complex were chosen in this work because of their representative pseudo spherical (icosahedral) and pseudo cylindrical symmetry.

### Basic Manipulations

#### Separation

With two separated coordinates files of the protein units as inputs, this tool would displace one of the two units in a user given distance away from the other. For clarification, one of the protein units would be fixed in its original coordinates and is referred as the fixed unit. The other protein unit would be manipulated to result in different positions and orientations and is hence referred as the manipulated unit.

The tool first calculate the mass center of both proteins, *C*_*fixed*_, and *C*_*manipulated*_, by averaging the coordinates of each individual atom after weighted by their corresponding atomic mass (Supplementary Equations 1, 2). With the obtained mass centers, a vector $\vec{M}$, form *C*_*fixed*_ to *C*_*manipulated*_ is calculated (Supplementary Equation 3). This vector $\vec{M}$ can then be normalized with its magnitude to obtain the vector $\vec{U}$ that defines the direction of the separation (Supplementary Equations 4, 5). With the vector $\vec{U}$ and the user-defined separation distance, d, a separation vector, $\vec{S}$, would then be generated (Supplementary Equation 6), which is then applied to the coordinates of the manipulated unit and create a new structure that is separated from the fixed unit by the user-defined distance *d* (Supplementary Equation 7).

#### Rotation

The rotation tool would rotate the manipulated unit around the fixed one by a user-given angle. This rotation operation can be carried out in xy plane (around z-axis), xz-plane or yz-plane as users prefer. Rotation in xy-plane is discussed here for a simplified demonstration.

The tool will start by calculating the mass center C_fixed_ using the method demonstrated in the section above. A vector, $\vec{M_{A}}$, from C_fixed_ to a randomly chosen atom A in the manipulated unit can be created (Supplementary Equation 8). The rotation vector, $\vec{R_{A}}$, would then be generated via multiplying vector $\vec{M_{A}}$ vector by a rotation matrix that included the user-defined angle for the desired rotation (Supplementary Equation 9). This obtained $\vec{R_{A}}$ is then applied on the x, y, z coordinates of the manipulated unit, generating a modified structure with the user-defined degree of rotation (Supplementary Equations 10, 11).

It is important to notice that the rotation of manipulated unit around the fixed one may introduce clashes if the atoms are closed to each other. Therefore, it is recommended to separate the manipulated unit from the fixed one to a proper distance upon using the rotation tool.

#### Spin

This tool allows the spinning of the manipulated unit with the respect to its own mass center. Similar to the rotation tool, the spinning can be performed in any of the *xy-, yx-*, or *xz-* plane. Spinning the protein unit in xy**-**plane is discussed here as a demonstration.

With the coordinate file of the unit to be manipulated as the input, this tool first calculates its mass center, C_manipulated_, using method discussed in section Separation. A vector, $\vec{M_{A}}$, from C_manipulated_ to a randomly chosen atom would then be generated and multiplied by a rotation matrix to generate the final spinning vector, $\vec{S_{A}}$, using the method demonstrated in section Rotation. The final coordinates of the atom will be calculated using the spinning vector. As the operation being carried out in the xy-plane, the z coordinate of each atom remind the same as original. This process would be repeated on each individual atom within the protein unit and output their spun coordinates into a separate file. To avoid clashes, it is recommended to separate the manipulated unit from the fixed one to a proper distance upon using the spinning tool.

#### Perpendicular Translation

The perpendicular translation tool shifts the manipulated unit along the line that is perpendicular to the vector of mass centers in the selected plane. The translation in xy-plane is shown as an example.

This tool calculates the mass centers of both protein units and the vector of mass centers, $\vec{M}$, the normalized vector $\vec{U}$, as well as the separation vector, $\vec{S}$, in a similar manner to that in section Separation (Supplementary Equations 12–15). The separation vector $\vec{S}$ would then be rotated 90Å or −90Å to generate the final translation vector $\vec{T}$ that contains information of the user-defined distance (Supplementary Equation 16). Finally, this tool modifies the coordinates of the manipulated unit using translation vector $\vec{T}$ to create a new structure which is translated along the line perpendicular to the mass center vector by a given distance d (Supplementary Equation 17). Similar to the rotation and spinning tool, it is also recommended to separate the manipulated unit from the fixed one to a proper distance upon using this tool in order to avoid any clashes.

### Capsid Structure Manipulation

#### Capsid Generation Tool

Many pdb files of multi-protein complexes deposited in Protein Data Bank (PDB) do not actually contain the coordinates of all the protein units within the complexes, making it inconvenient for researchers who study protein-protein interactions among multiple protein units. However, instructions on how to construct the missing units from the given units are given as BIOMT matrices (Table 1). Within the BIOMT matrices, the numbers of biomolecule to be constructed (Table 1, Column3), as well as the corresponding transformation matrices are provided (Table 1, Column4–7). Therefore, in order to generate the structure of all the individual biomolecule unit within the pseudo spherical (icosahedral) viral capsid, an input coordinate file containing BIOMT matrices information is required.

**Table 1:** Demonstration of the BIOMT matrices provided in PDB files.

**Column1**	**Column 2**	**Column 3**	**Column 4**	**Column 5**	**Column 6**	**Column 7**
REMARK 350	BIOMT1	N	*a*	*b*	*c*	*d*
REMARK 350	BIOMT2	N	*e*	*f*	*g*	*h*
REMARK 350	BIOMT3	N	*i*	*j*	*k*	*l*

As shown in Table 1, each transformation matric contains BIOMT1, BIOMT2, BIOMT3, which would be apply on x, y, and z coordinates, respectively, using the following equations:

(1) $$\begin{array}{l} \left\{ \begin{array}{c} x_{f}=ax_{0}+by_{0}+cz_{0}+d\\ y_{f}=ex_{0}+fy_{0}+gz_{0}+h\\ z_{f}=ix_{0}+jy_{0}+kz_{0}+l \end{array}\right. \end{array}$$

Where the coefficients *a* to *l* are provided by the BIOMT matrices (Table 1), and *x*_0_, *y*_0_, and *z*_0_ represent the original coordinates of individual atom in the given molecule. The calculation would be performed on all other atoms until the structure of protein unit is completed and output as a separated file. This process will then repeat with the next BIOMT matrix until all the required protein units are generated (Figures 1A,B).

This tool can be applied in generating structures of individual protein units form any multi-protein complex as long as the BIOMT matrices are provided. As a demonstration, the initial structure of the TCV capsid was downloaded from PDB (ID 3ZX8), by which the structure of a capsomer and the BIOMT matrices were provided. Using the capsid generation tool, 60 copies of capsomer structures were generated and assembled into the native structure of TCV capsid (Figure 1B). These capsomer structures can then be collected for further studies, where the interactions among capsomers are investigated.

#### Capsid Expansion Tool

The next tool allows the shifting of all capsomers away from the mass center of the whole capsid resulting in a viral capsid expended by a user desired distance (Figure 1C).

The first step in this operation is to find the capsid's mass center, C_capsid_, which would be done in a similar manner to the capsid generation tool. When determining the mass center of TCV capsid, this tool first calculates the mass center of the primary capsomer, and then transformed the obtained coordinates according to the given BIOMT matrices. Sixty copies of coordinates would be generated and presenting the mass center of corresponding capsomers in the TCV capsid. With these coordinates, the coordinates of the mass center of the whole TCV capsid can then calculated by averaging the mass center coordinates of its individual capsomer, as shown in the following equation:

(2) $$\begin{array}{l} \left\{ \begin{array}{c} C_{capsid}\left(x\right)=\frac{\sum_{n=1}^{N}x_{n}}{N}\\ C_{capsid}\left(y\right)=\frac{\sum_{n=1}^{N}y_{n}}{N}\\ C_{capsid}\left(y\right)=\frac{\sum_{n=1}^{N}z_{n}}{N} \end{array}\right. \end{array}$$

Where *N* is the total number of capsomers, *x*_*n*_, *y*_*n*_, and *z*_*n*_ are the coordinates of the mass center of the capsomer *n*.

Next, the tool generates the 60 copies of vectors, ${\vec{S}}_{n}$, from the mass center of the whole capsid to the mass center of individual capsomer by the following equation; The vectors, ${\vec{S}}_{n}$, would define the direction of the shifting the individual capsomers.

(3) $$\begin{array}{l} \left\{ \begin{array}{c} {\vec{s}}_{n,x}=C_{capsid}\left(x\right)-C_{N}\left(x\right)\\ {\vec{s}}_{n,y}=C_{capsid}\left(y\right)-C_{N}\left(y\right)\\ {\vec{s}}_{n,z}=C_{capsid}\left(z\right)-C_{N}\left(z\right) \end{array}\right. \end{array}$$

where *C*_*capsid*_*(x), C*_*capsid*_*(y)*, and *C*_*capsid*_*(z)* are given in Equation (2), *C*_*N*_*(x), C*_*N*_*(y)*, and *C*_*N*_*(z)* are the coordinates of mass center of the capsomer *n*.

The shifting (expansion) distance can again be defined by user. To make it more convenient of users who don't have direct measurement of the desired distance, we introduce the concept of a mean radius of mass distribution, the mean mass radius R_M_, which is defined using the following equation:

(4) $$\begin{array}{l} \left\{ \begin{array}{c} r_{i}=\sqrt{{\left[C_{capsid}\left(x\right)-x_{i}\right]}^{2}+{\left[C_{capsid}\left(y\right)-y_{i}\right]}^{2}+{\left[C_{capsid}\left(z\right)-z_{i}\right]}^{2}\;}\\ R_{M}=\frac{\sum_{i=1}^{I}m_{i}r_{i}}{\left|M_{T}\right|} \end{array}\right. \end{array}$$

Where *x*_*i*_, *y*_*i*_, and *z*_*i*_ are the coordinates of atom *i, r*_*i*_ is the distance between a single atom *i* and the mass center of the capsid *C*_*capsid*_, m_i_ is the atomic mass of the corresponding atom, and *M*_*T*_ is the total atomic mass of all atoms within the capsid.

Thanks to the icosahedral symmetry of the vial capsid, the distances from *C*_*capsid*_ to the mass center of individual capsomer are equal. The calculation of the *R*_*M*_ can be simplified to one step using following equation:

(5) $$\begin{array}{l} R_{M}={|\vec{S}}_{n}|=\sqrt{{\left[C_{capsid}\left(x\right)-C_{N}\left(x\right)\right]}^{2}+{\left[C_{capsid}\left(y\right)-C_{N}\left(y\right)\right]}^{2}+{\left[C_{capsid}\left(z\right)-C_{N}\left(z\right)\right]}^{2}\;} \end{array}$$

where only the coordinates of the capsid mass center *C*_*capsid*_*(x, y, z)*, and that of the one capsomer *n* is needed.

With the given distance, *d*, a expansion vector, $\vec{E}$, would then be generated using by the following expressions:

(6) $$\begin{array}{l} \left\{ \begin{array}{c} {\vec{E}}_{x}=d\cdot R_{M}\cdot {\vec{S}}_{n,x}\;\\ {\vec{E}}_{y}=d\cdot R_{M}\cdot {\vec{S}}_{n,y}\\ {\vec{E}}_{z}=d\cdot R_{M}\cdot {\vec{S}}_{n,z} \end{array}\right. \end{array}$$

Finally, the tool generates the expanded structures based on the given primary capsomer, BIOMT matrices and the calculated expansion vector $\vec{E}$, using the following equation:

(7) $$\begin{array}{l} \left\{ \begin{array}{c} x_{f}=ax_{0}+by_{0}+cz_{0}+d+{\vec{E}}_{x}\\ y_{f}=ex_{0}+fy_{0}+gz_{0}+h+{\vec{E}}_{y}\\ z_{f}=ix_{0}+jy_{0}+kz_{0}+l+{\vec{E}}_{z} \end{array}\right. \end{array}$$

Where the coefficients *a* to *l* are provided by the BIOMT matrices (Table 1), and *x*_0_, *y*_0_, and *z*_0_ are the coordinates of individual atom in the primary capsomer. The calculation would be repeat on all atoms within the capsomer and output the expanded coordinates into a separated file. This process then goes on with the next BIOMT matrix until the expanded capsid is generated (Figures 1B,C).

As a demonstration, a TCV capsid expended by 0.5R_M_ was generated by shifting the individual capsomers 0.5R_M_ away from the mass center of TCV capsid (Figure 1C). With the structures of expended capsid and its individual capsomer, investigation that aims to determine the driving force of intact viral capsid assembly, can be carried out as discussed in the later section.

#### Capsomer Detachment Tool

This tool detaches a single capsomer from the viral capsid by a user defined distance. Compare to previous tools, this one will output a structural file of the shifted capsomer and the rest of the capsid in two separated files. The work flow of this tool is very similar to that of the expansion tool, except the expansion vector would only be applied on the chosen capsomer. The detachment distance can be user defined relatively to the mean mass radius R_M_. As a demonstration, one of the capsomer from TCV capsid was separated from the rest of the capsid from 0.4R_M_ to 1.0R_M_ in 0.1R_M_ intervals (Figure 1D).

Once the structures of the detached capsomer and the rest of the capsid are obtained, operations including spinning and rotation, can then be carried out for the purpose of investigating the interactions between the one capsomer and rest of the capsid during viral capsid assembly. After detached from the TCV capsid by 0.7R_M_, the capsomer was spun in the xy-plane (around z-axis) from −90Å to 90Å in 2Å interval (Figure 1E) using the spinning tool described in section Spin. The capsomer were also rotated around the rest of the capsid from −20Å to 20Å in 2Å interval (Figure 1F) using the rotation tool described in section Rotation.

By detaching, spinning and rotating a single capsomer, different orientations and distances of capsomer with respect to the rest of the capsid were obtained, which can be used for the capsid assembly studies where the driving force can be investigated in a manner of mimicking the dynamic assembly process.

#### Cylindrical Structure Manipulation

**Figure 2:**
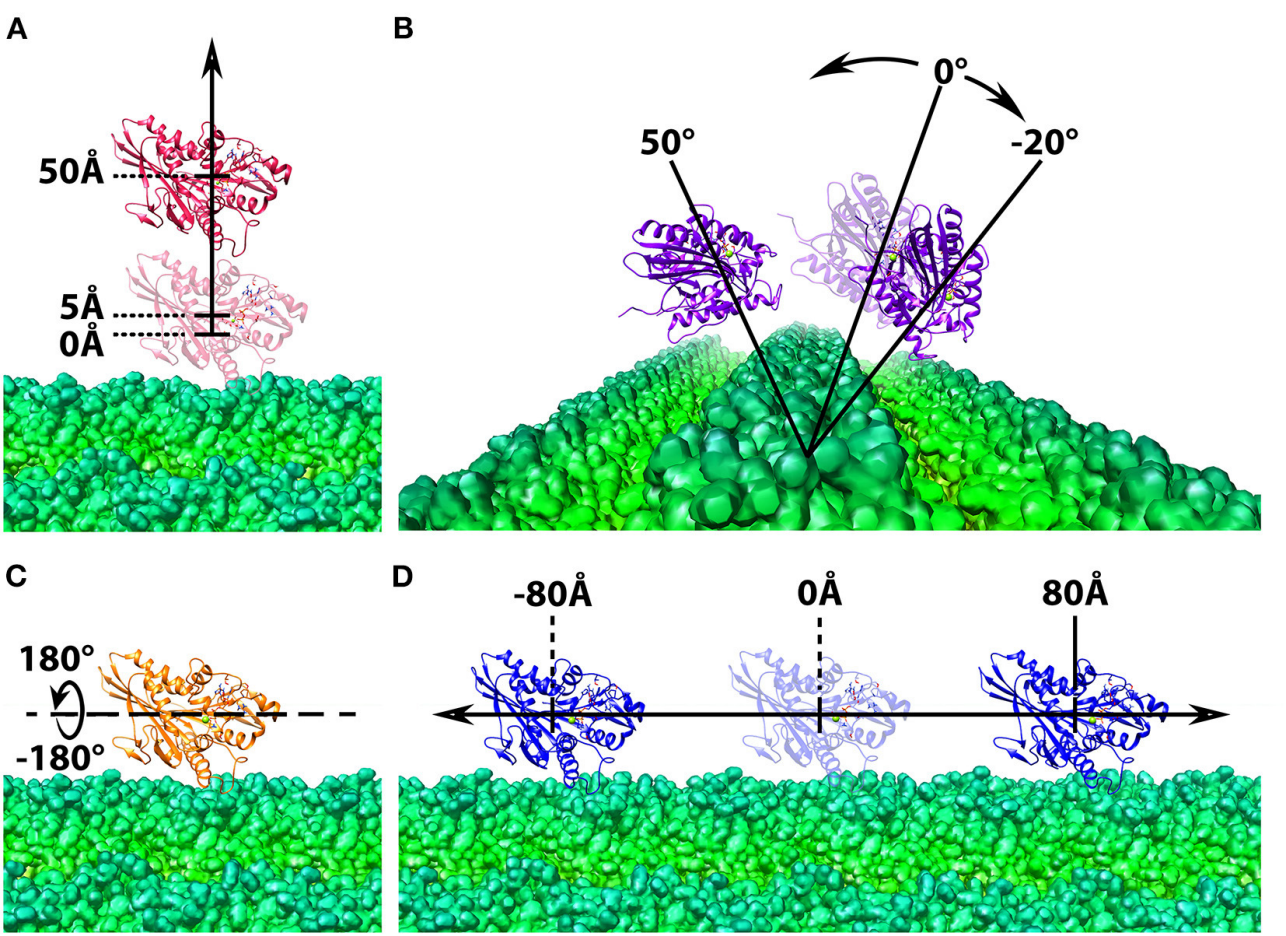
Structure manipulation of the kinesin with respect to microtubule. In each of the four operations, the microtubule is shown at the bottom in green and shown in density map generated by chimera (Pettersen et al., 2004; Goddard et al., 2007), whereas the motor domain of kinesin is shown on the top in ribbon with various color, red, purple, orange, and blue. The motor domain is manipulated by **(A)** shifted from the microtubule from 5Å to 50Å in 2Å interval; **(B)** rotated around one chosen microtubule unit form −20Å to 50Å in 2Å interval; **(C)** spun from −180Å to 180Å in 2Å interval around the z-axis; **(D)** translated along the microtubule from −80Å to 80Å in 2Å interval.

The application of the manipulating tool can be extended in complexes where cylindrical structures are involved. One example is to mimic the kinesin motor's movement on the microtubule filament, which can be subsequently used in the investigation of kinesin-microtubule interactions during the cargo transportation. In this work, the complex structure of kinesin binding with microtubule is generated and described in our previous paper (Li et al., 2016b). However, the StructureMan tool can be used to any other microtubule and cylindrical biomolecules. With the tools described above, 4 different operations of the kinesin motor domain were performed: (1) shifted away from the microtubule by 5Å to 50Å in 2Å interval using the separating tool (Figure 2A); (2) rotated around one chosen microtubule unit from −20Å to 50Å in 2Å interval using the rotation tool (Figure 2B). This rotation range was limited by the steric effects of the neighboring microtubule units; (3) spun from −180Å to 180Å in 2Å interval around the Z axis using the spin tool (Figure 2C). (4) translated along the microtubule from −80Å to 80Å in 2Å interval using the perpendicular translation tool (Figure 2D); In each operation, the structures of kinesin motor domain with various distances and orientations were output separately and collected for DelPhi (Li et al., 2012a,b, 2013b) and DelPhiForce (Li et al., 2017a,b,c) calculation.

### Electrostatic Potential Calculations by DelPhi

**Figure 3:**
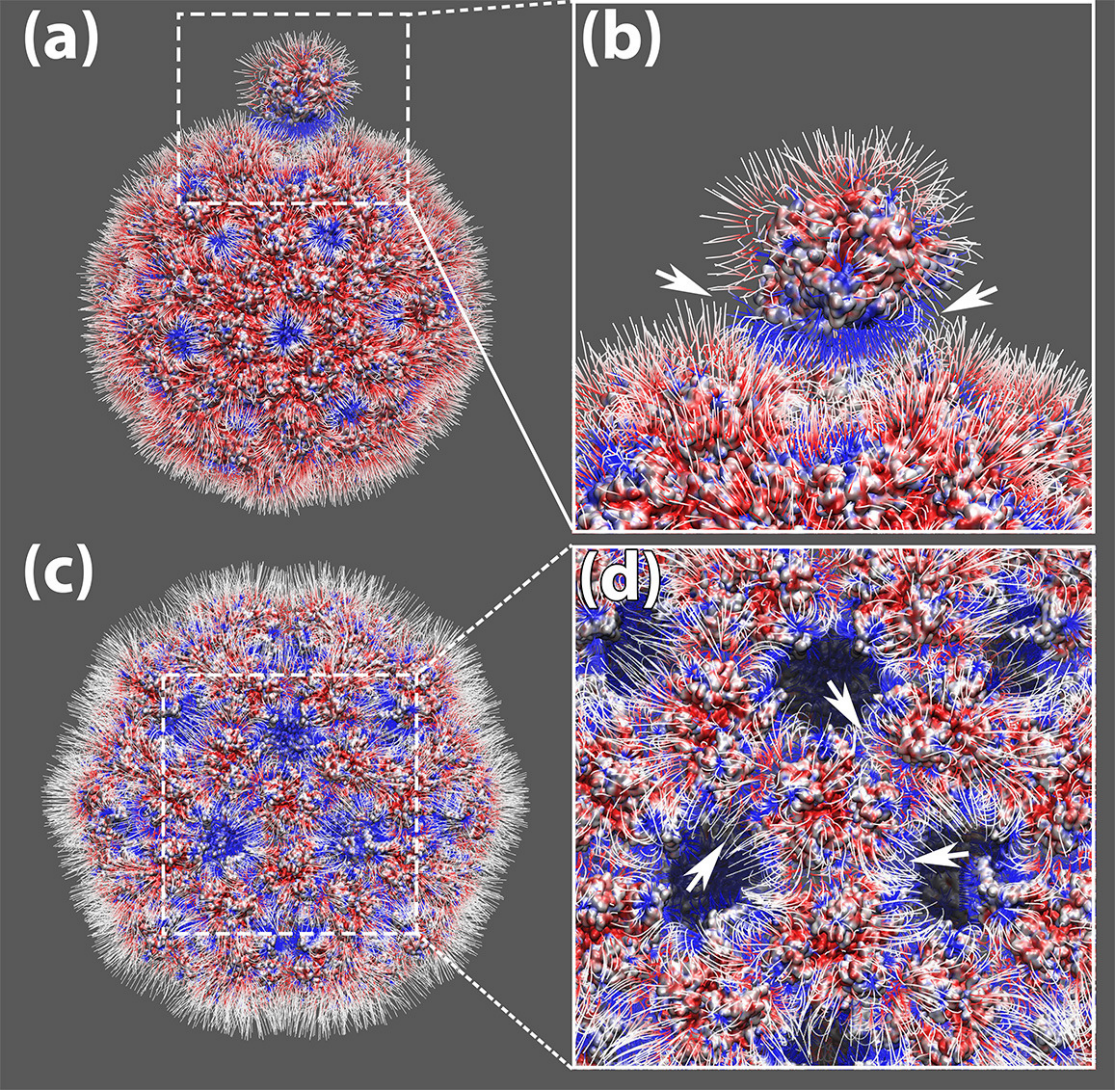
The electrostatic potential field lines among capsomers of TCV capsid. **(a)** The electrostatic potential field lines of a capsomer detached away by 0.5R_M_ and the rest of the capsid. **(b)** Zoom-in area of the electrostatic potential field lines between the detached capsomer and the rest of the capsid, where their attractive interaction ware pointed out by white arrows; **(c)** The electrostatic potential field lines of the expended capsid obtained by shifting individual capsomers 0.5RM away from the mass center of the whole capsid. **(d)** Zoom-in area of the electrostatic potential field lines among the expanded capsid. The attractive interaction around one capsomer are pointed out by white arrows; All four panels were rendered by VMD (Humphrey et al., 1996). All capsomer surfaces are generated using the “Quicksurf” method. Negatively and positively charged capsomer surface areas are colored from red to blue with a scale of −3.0 to 3.0 kT/Å. The electric field lines were also colored using the same color scheme.

Electrostatic calculations were performed on the complex of detached capsomer and incomplete TCV capsid as well as the expended capsid collected from previous sections using method described in our previous paper (Xian et al., 2019). The electrostatic potentials as well as the interactions among the capsomers are visualized in Visual Molecular Dynamics (VMD) (Humphrey et al., 1996; Figure 3). The surfaces of the capsid and capsomers are generated using the “Quicksurf” method in VMD and colored from red to blue in a scale range of −3.0 to 3.0 kT/Å. More information on DelPhi analysis can be accessed through this tutorial: http://compbio.clemson.edu/delphi.

### Electrostatic Binding Forces Calculation by DelPhiForce

**Figure 4:**
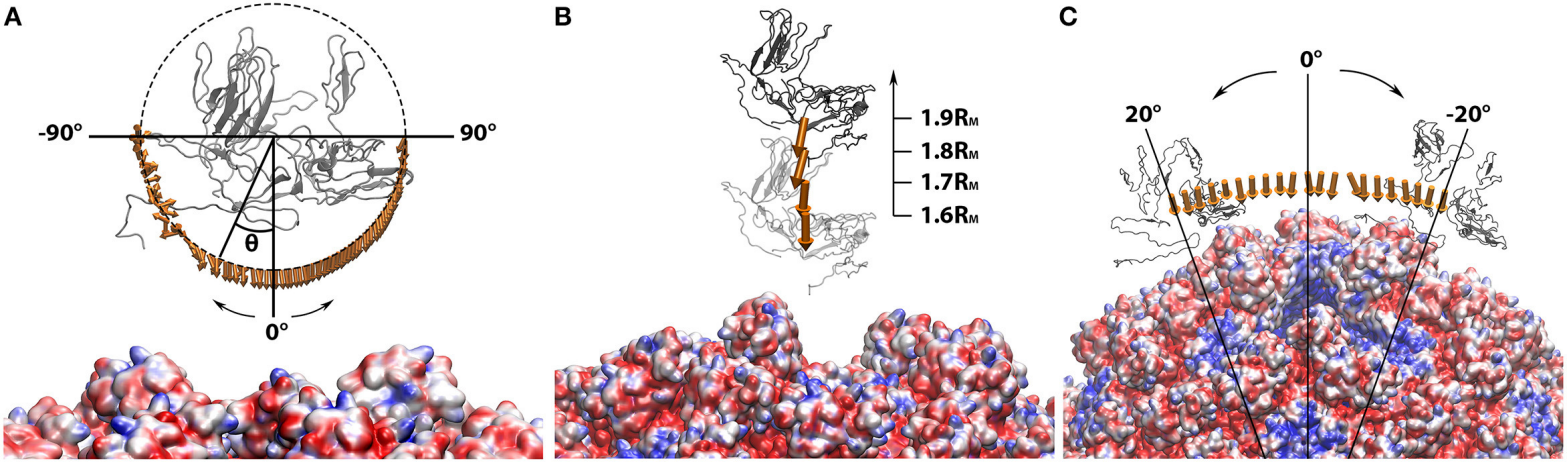
Electrostatic forces between the manipulated capsomer and the rest of the capsid while the capsomer was **(A)** separated from the rest of the capsid by 0.4R_M_ to 0.7R_M_ in 0.1R_M_ interval; **(B)** spun around the z-axis from −90Å to 90Å in 2Å interval; **(C)** rotated around the rest of the capsid from −20Å to 20Å in 2Å interval. In all three panels, the manipulated capsomer was shown in gray ribbon. The rest of the capsid shown in “Quicksurf” colored from red to blue in a scale of −3.0 to 3.0 kT/Å. The electrostatic forces are represented by arrows. In order to clearly show the directions of all binding forces, the arrows in each panel ware normalized to the same size. The tails of arrows in **(B,C)** ware placed at the mass centers of the manipulated capsomer. In **(A)**, the arrow tails are place on a circle where the spinning degrees can be differentiated by the angle theta (θ). All images are rendered by VMD.

To examine the roles of electrostatic interactions in the process of viral capsid assembly, 115 structures of viral capsomers in various orientations and distances were collected from previous sections and prepared for DelPhiForce calculations using the method mentioned in our previous paper (Xian et al., 2019). The calculated electrostatic forces between the manipulated capsomer and the rest of the capsid were visualized and in VMD (Humphrey et al., 1996), where they are represented by arrows. The rest of the capsids are shown using the “Quicksurf” method and colored from red to blue in a scale range of −3.0 to 3.0 kT/Å (Figure 4).

**Figure 5:**
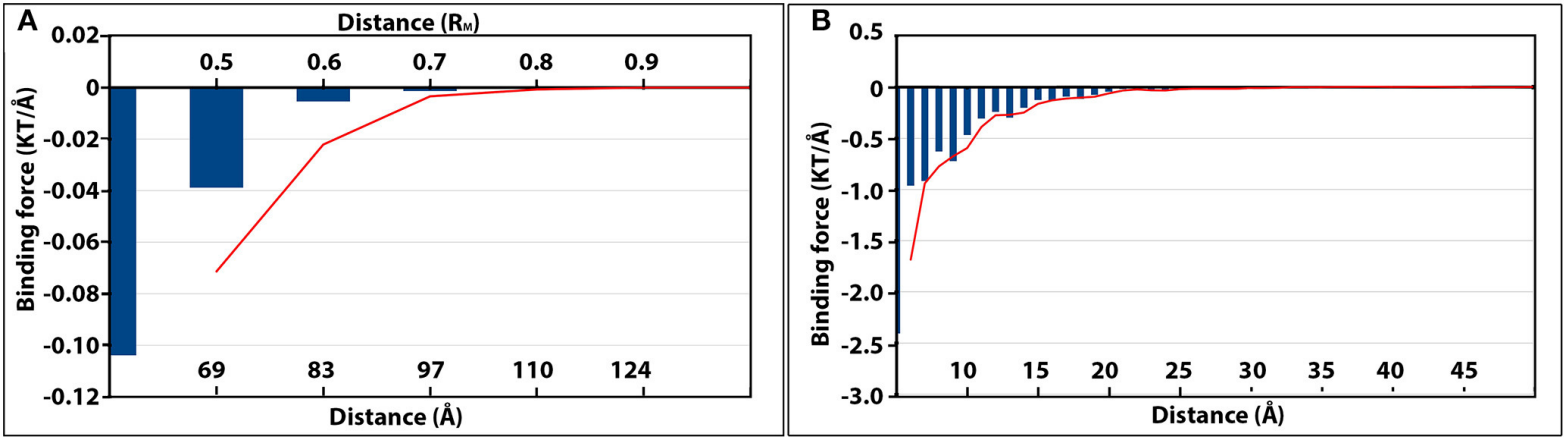
The magnitudes of electrostatic binding forces between the detached capsomer and incomplete TCV capsid **(A)**, and the kinesin motor domain and microtubule **(B)**. In both panels, the strength of the binding forces is represented by the blue vertical histogram bars. The more negative value of binding force (KT/Å), the stronger attractive electrostatic binding force. The changes of binding force as the separation distance increased were shown by the red moving average trendlines in both panels.

In order to underline the significances of electrostatic interactions in driving kinesin's movement along the microtubule, 342 structures of microtubule and kinesin motor domain in different orientations and distances were collected for DelPhiForce calculations. The parameters for these calculations were set as the same as those of TCV capsid. The visualization of the electrostatic forces was also done in VMD using the same method, except the surface of microtubule were obtained by the “Surf” option in VMD (Figure 5). More information on DelPhiForce analysis can be accessed through this tutorial: http://compbio.clemson.edu/delphi-force-web.

## Results and Discussion

### Electrostatic Potential of the TCV Capsomers and Its Capsid

The charge distribution on capsomers mainly has two functions: First, the electrostatic interactions among capsomers play significant roles in assembling and stabilizing the whole capsid structure (Li et al., 2012a; Salas et al., 2019; Xian et al., 2019). Second, electrostatic interactions between capsid and DNA/RNA stabilize the encapsidated genomic materials by neutralizing the repulsive forces between the DNA/RNAs (Bakker et al., 2012). In this work, we mainly focused our investigations on electrostatic interactions among capsomers of TCV using the StructureMan.

The electrostatic potential calculations from DelPhi demonstrated the charge distribution on the inner and outer surface of the viral capsid. The inner surface of the viral capsid is dominated by positive charges as shown in Figures 3c,d, which explains why the capsomers play crucial roles in stabilizing the packed genomic RNA in previous studies.

On the outer surface of the viral capsid, negatively charged residues are distributed rather evenly through the whole capsid, while the positively charged residues are mostly located at 5-fold axis and 3-fold axes (Figure 3a). After one chosen capsomer is detached from the rest of the capsid by 0.5R_M_ (50 percent of the particle mean mass radius), strong attractive electric field lines are present between the detached capsomer and the rest of the capsid (Figure 3b), suggesting that the electrostatic interaction guides capsomers to build the viral capsid.

To study the overall electric field lines among the capsomers for a whole capsid, the StructureMan was utilized to expand the capsid. This “expand” operation shifted each of the capsomers away from the rest of the capsid (Figures 3c,d). Electrostatic calculation of the expended capsid shown the electric field lines located at the interfaces of capsomers (Figures 3c,d). High density of electric field lines were found among individual capsomers, indicating the present of strong attractive interactions (Figure 3d). These attractive interactions were found throughout the viral capsid, which demonstrated the role of electrostatic interactions in stabilizing the whole viral capsid.

### Electrostatic Binding Force Between One Detached Capsomer and the Rest of the Capsid

To further characterize the role of electrostatic interaction in the vial capsid assemble process, the electrostatic binding forces were calculated using DelPhiForce (Li et al., 2017a,b). With the structure generated from the StructureMan, the electrostatic binding forces were studied in various orientation and distances of the capsomers (Figure 4).

While one chosen capsomer was detached from the rest of the capsid, DelPhiForce was utilized to calculate the electrostatic binding forces, which were represented by orange arrows in Figure 4A. Note that all the force arrows are normalized to the same size in order to demonstrate their directions clearly. To compare the strengths of these binding forces, the magnitudes of these binding forces were plotted against the distance between the detached capsomer and its native position (Figure 5A), where the more negative value represents the stronger attractive binding force. Binding forces data at 0.3R_M_ or less were not considered because of the possibility of clashes between the atoms. The binding force became neglectable after the capsomer was detached by 0.8R_M_ or further (Figure 5A). The forces within the range of 0.4R_M_ to 0.7R_M_ are all attractive as the arrows point toward the rest of the capsid (Figure 4A). This indicated that the effective range of electrostatic forces between the capsomer and the rest of the capsid is about 0.7R_M_(~97Å), which suggests that the electrostatic binding forces guide capsomers from long distance during the viral capsid assembling process.

The binding forces between the detached capsomer and the rest of the capsid were also calculated while the detached capsomer was spun (visualized in Figure 4B). If the capsomer was rotated around z-axis within the range from −45Å to 90Å, the binding forces remained attractive. When the capsomer was rotated from −90Å to −45Å, the binding force became repulsive due to the effect introduced by putting the wrong-orientated bulky S domain too close to the rest of the capsid, which resulted in strong electrostatic repulsive forces. This suggests that the electrostatic forces contribute in adjusting the orientations of the capsomers to the native orientations, which were more electrostatically favorable compared to the non-native orientations.

Previous studies on viral capsids have demonstrated the interactions between an individual capsomer and its adjacent capsomers are crucial in the capsid assembly process (Salas et al., 2019; Xian et al., 2019). Here we focused on the interaction between one capsomer and the rest of the capsid. As the detached capsomer was rotated around the rest of the capsid using the StructureMan, the binding forces were analyzed (Figure 4C). The rotation was carried out within a range that the detached capsomer was still relatively close to the cavity created by capsomer detachment. As the arrows are all orientated toward the capsid, we conclude that the electrostatic interaction is again attractive between the detached capsomer and the rest of the capsid.

### Electrostatic Binding Force Between Kinesin Motor Domain and Microtubule

**Figure 6:**
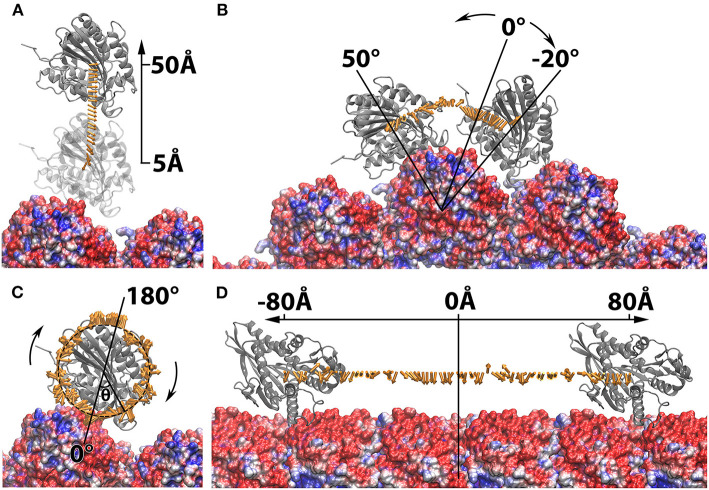
Electrostatic forces between the manipulated kinesin motor domain and microtubule while the kinesin motor domain is **(A)** separated from the microtubule from 5Å to 50Å in 2Å interval; **(B)** rotated around one chosen microtubule unit form −20Å to 50Å in 2Å interval; **(C)** spun from −180Å to 180Å in 2Å intervals around the z-axis. **(D)** Translated along the microtubule from −80Å to 80Å in 2Å interval. The kinesin motor domains are shown in ribbon. The microtubule are shown in surface colored from red to blue in a scale of −3.0 to 3.0 kT/Å. The electrostatic forces are represented by arrows. In **(C)**, the arrow tails are place on a circle where the spinning degrees can be differentiated by the angle theta (θ), while the tails of arrows in all other panels are placed at the mass centers of the manipulated capsomers. The force arrows in each panel are normalized to the same size. All images are rendered by VMD.

Similar analyses of the electrostatic binding forces were performed with the kinesin-microtubule complex in which the kinesin motor domain was manipulated by various orientations and distances (Figures 5B, 6). As the kinesin motor domain was separated from the microtubule, the strengths of the attractive binding forces reduced and became insignificant when the distance reached 25Å (Figures 5B, 6A). When the kinesin was separated from the microtubule less than 15Å, the electrostatic binding forces were exerted toward the native binding site on the microtubule. When the separation was in the range of 15Å−25Å, the binding forces were orientated to the neighboring binding site. This suggests that as the distance between the kinesin motor domain and the microtubule increases, the binding force toward the neighboring binding site becomes competitive to that toward the native binding site.

While the kinesin was rotated around one microtubule within the range of −16Å to 10Å, the binding force was exerted toward the native binding site (Figure 6B). If the kinesin was rotated further than −16Å, the force became repulsive due to the steric effect from the neighboring microtubule units. When the kinesin was rotated to the top of the microtubule units, the binding force first became repulsive and then changed to attractive as it traveled close enough to the neighboring native binding site (Figure 6B). When the kinesin motor domain was spun from −10Å to 10Å (the motor is still in near-native orientation), the binding forces remain attractive. However, when the orientation of kinesin motor domain is significantly changed, the binding forces became randomly directed (Figure 6C). The results from both rotation and spin operations reveal that the electrostatic interaction favors the native or near-native orientations of kinesin motor domain, and when its orientation is altered, the binding force reorient it to the native orientation by providing a repulsive binding force.

In the operation where the kinesin motor domain was translated along the microtubule, the binding forces were consistently exerted toward the native binding side within the range of −26Å−12Å (Figure 6D). While traveling between the native binding site to the neighboring binding site, the binding forces were shown to be mostly repulsive. However, as the kinesin motor domain traveled closer to the neighboring binding site (from −70Å to −80Å, and 60Å to 80Å), the binding forces were again orientated toward the neighboring binding sites. These results demonstrate that the electrostatic interactions make a significant contribution in guiding the kinesin by favoring the binding on the native binding sites and rejecting the non-native binding positions. Our discovery on the electrostatic interaction between kinesin and microtubule can explain and support the thermal ratchets model for kinesin's motility (Magnasco, 1993; Hwang and Karplus, 2019). The electrostatic analyses in this study also match the previous results from Brownian simulations for kinesins (Grant et al., 2011).

## Conclusion

Understanding biomolecular interactions is crucial and fundamental to study the biology problems. Due to their large scales, many biomolecular interactions are difficult to be studied via all atom simulations. Here we introduce a Structure Manipulation tool (StructureMan) to offer comprehensive operations for the structures in large scale biomolecular interactions, such as interactions in the viral capsid and molecular motor-microtubule complexes. This StructureMan tool contains operations which can be utilized to study the interactions in large biological systems. Combining with electrostatic calculation tools such as DelPhi and DelPhiForce, StructureMan can be used to reveal the detailed biomolecular interactions. Two examples are demonstrated in the results and discussion section, which show that the StructureMan is beneficial when studying the biomolecular interactions in large scale biomolecular complexes.

There are four basic and two advanced operations developed in the StructureMan. Note that these basic operations in StructureMan are different from those in existing visualization programs. Those tools in the existing visualization programs can shift or rotate a single biomolecule; However, these four basic operations of StructureMan take two biomolecules as inputs and do the operations between the two biomolecules. For examples, the separation tool in StructureMan shifts the ligand from the receptor in the direction of their mass center connection line, while the existing tools can only shift a single protein. If users want to shift a ligand from the receptor in the direction of their mass center connections, users need to calculate the shifting vector first and normalize the vector, then use the existing tools to shift the ligand. The rotation operation in StructureMan rotates the ligand around the mass center of the receptor. This operation cannot be easily achieved by the existing visualization programs. Instead, users need to write some script to complete such an operation. Advanced operations in StructureMan are even more comprehensive. For example, the capsid expansion operation takes one capsomere as input structure and generates a structure of expanded capsid in which every two adjacent capsomers are separated by a distance defined by the users. Such an operation is not in any of the existing tools. Another advantage is that the StrucrueMan is written in shell script, which can be easily used to handle large number of structures (such as a big number of frames from MD simulations).

In this work, we first focused on investigations on electrostatic interactions among capsomers of TCV using the StructureMan. The charge distribution on the inner surface of the viral capsid indicates that the capsomers stabilize the packed genomic RNA, as observed in previous studies. On the outer surface of the viral capsid, strong attractive electric field lines imply that the electrostatic interactions guide capsomers to build the viral capsid. The “expand” operation shifted each of the capsomers away from the rest of the capsid, which reveals that the attractive interaction among the capsomers is a key factor to stabilize the whole viral capsid.

The StructureMan and DelPhiForce were utilized to further characterize the electrostatic binding forces in the viral capsid. Results demonstrate that the effective range of electrostatic forces between the capsomer and the rest of the capsid is about 0.7R_M_ (~97Å), which suggests that the electrostatic binding forces guide capsomers from long distances in the viral capsid assembling process. The spin and rotation operations in the StructureMan show that the electrostatic forces contribute in adjusting the orientations of the capsomers to the native orientations.

Similar analyses of the electrostatic binding forces were performed to the kinesin-microtubule complex, where the kinesin motor domain was manipulated by various orientations and distances. The results suggest that when the distance between the kinesin motor domain and the microtubule increases, the binding force toward the neighboring binding site becomes competitive to that toward the native binding site. The calculations from both rotation and spin operations reveal that the electrostatic interaction favors the native or near-native orientations of kinesin motor domain. When the orientation of kinesin motor domain is altered, the binding force reorients it to the native orientation by providing a repulsive binding force. In the operation where the kinesin motor domain was translated along the microtubule, the calculations demonstrate that the electrostatic interactions make significant contributions in guiding the kinesin by favoring the binding on the native binding sites and rejecting the non-native binding positions.

Besides the two examples demonstrated in this work, the StructureMan program is able to help the researchers to study many other large-scale biomolecular interactions. We expect the StructureMan to be combined with DFMD method (Peng et al., 2019) to investigate the biomolecular interactions in the perspective of molecular dynamic simulations in our future work. This novel tool provides an alternative approach to study the biomolecular interactions, especially for large scale biology problems. The StructureMan tool is available at our website: http://compbio.utep.edu/static/downloads/script-for-munipulation2.zip.

## Data Availability Statement

The raw data supporting the conclusions of this article will be made available by the authors, without undue reservation.

## Author Contributions

LL and WQ proposed the idea, conducted entire research, and wrote the manuscript. YXia and YXie run the calculations, collected the data, analyzed the results, and wrote the manuscript. SS developed and tested the programing code. CK tested the program and revised the manuscript. All authors contributed to the article and approved the submitted version.

## Conflict of Interest

The authors declare that the research was conducted in the absence of any commercial or financial relationships that could be construed as a potential conflict of interest.
