# Supplementary material for: StructureMan: A Structure Manipulation Tool to Study Large Scale Biomolecular Interactions
Source: Front Mol Biosci. 2021 Jan 11;7:627087. doi: 10.3389/fmolb.2020.627087 (PMC7831659; doi:10.3389/fmolb.2020.627087)
Supplement: Supplementary file 1 [file Data_Sheet_1.DOCX]

Supplementary Material

# Basic manipulations

# Separation

The tool will first calculate the mass center of both protein units through eq. 1, where the center of mass *C* of the protein unit is calculated by averaging the coordinates of each individual atom after weighted by their corresponding atomic mass:

|  | $\left\{ \begin{aligned} C_{x}= \frac{\sum_{n=1}^{N} m_{n}r_{n}\left( x \right)}{M_{T}} \\ C_{y}=\frac{\sum_{n=1}^{N} m_{n}r_{n}\left( y \right)}{M_{T}} \\ C_{z}=\frac{\sum_{n=1}^{N} m_{n}r_{n}\left( z \right)}{M_{T}} \end{aligned} \right.$ | (1) |
| --- | --- | --- |

where *N* is the total number of atoms in the system; *r_n_(x), r_n_(y)* and *r_n_(z)* are the coordinates of an atom, *n*; *m_n_* is the atomic mass of atom *n*; *M_T_* is the total atomic mass of all atoms within the protein unit and can be calculated using the following equation:

|  | $M_{T}=\sum_{n=1}^{N} m_{n}$ | (2) |
| --- | --- | --- |
|  |  |  |

With the obtained mass centers *C_fixed_* , and *C_manipulated_* , a vector $\vec{M}$, form *C_fixed_* to *C_manipulated_* would then be create using the following expression:

|  | $\left\{ \begin{aligned} \vec{M}_{x}=C_{manipulated}\left( x \right)-C_{fixed}(x) \\ \vec{M}_{y}=C_{manipulated}\left( y \right)-C_{fixed}(y) \\ \vec{M}_{z}=C_{manipulated}\left( z \right)-C_{fixed}(z) \end{aligned} \right.$ | (3) |
| --- | --- | --- |

And its magnitude can be calculated:

| $\vert\vec{M}\vert=\sqrt{{(\vec{M}_{x})}^{2}+{(\vec{M}_{y})}^{2}+{(\vec{M}_{z})}^{2}}$ | | (4) | |
| --- | --- | --- | --- |
| The $\vec{M}$ can then be normalized with its magnitude to obtain the the vector $\vec{U}$ that defines the direction of the separation: | |  | |
|  | $\left\{ \begin{aligned} \vec{U}_{x}=\frac{\vec{M}_{x}}{\left\vert\vec{M} \right\vert} \\ \vec{U}_{y}=\frac{\vec{M}_{y}}{\left\vert\vec{M} \right\vert} \\ \vec{U}_{z}= \frac{\vec{M}_{z}}{\left\vert\vec{M} \right\vert} \end{aligned} \right.$ | | (5) |
|  |  | |  |

With the given separation distance, **d**, a separation vector, $\vec{S}$, would then be generated using by the following expressions:

| $\left\{ \begin{aligned} \vec{S}_{x}=d\cdot\vec{U}_{x} \\ \vec{S}_{y}=d\cdot\vec{U}_{y} \\ \vec{S}_{z}=d\cdot\vec{U}_{z} \end{aligned} \right.$ | (6) |
| --- | --- |

The final step in this tool will be modify the coordinates of the manipulated unit to create a new structure which is separated from the fixed unit by a user given distance *d* using the following equation:

| $\left\{ \begin{aligned} x_{f}=x+\vec{S}_{x} \\ y_{f}=y+\vec{S}_{y} \\ z_{f}=z+\vec{S}_{z} \end{aligned} \right.$ | (7) |
| --- | --- |

Where *x_f_, y_f_* and *z_f_* represent the final coordinates of each individual atom within the manipulated unit. These new coordinates would be output into a separated structural file where all the other non-coordinate information would be kept the same as input.

# Rotation

The tool will start by calculating the mass center C_fixed_ using the method demonstrated in the section above. A vector,$\vec{M_{A}}$, from C_fixed_ to a randomly chosen atom A in the manipulated unit can be created using following equation:

| $\left\{ \begin{aligned} \vec{M}_{Ax}=x_{A}-C_{\mathrm{fixed}}(x) \\ \vec{M}_{Ay}= y_{A}-C_{\mathrm{fixed}}(y) \end{aligned} \right.$ | (8) |
| --- | --- |

Where *x_A_* and *y_A_* are the coordinates of the chosen atom. Note that the z coordinated would be kept as the original value since the rotation is operated in the xy-plane.

The rotation vector, $\vec{R_{A}}$, of the chosen atom would then be generated via multiplying vector $\vec{M_{A}}$ vector by a rotation matrix as the following equation:

| $\vec{R_{A}}=\left[ \begin{matrix} cos(\theta) & -sin(\theta) \\ sin(\theta) & cos(\theta) \end{matrix} \right]\left[ \begin{matrix} \vec{M}_{Ax} \\ \vec{M}_{Ay} \end{matrix} \right]$ | (9) |
| --- | --- |

Where *θ* is the desired rotation angle input by the user. Hence, the x, y component of the $\vec{R_{A}}$ vector can be calculated as follow:

| $\left\{ \begin{aligned} \vec{R}_{Ax}=\vec{M}_{Ax}\cdot\cos\left( \theta\right)-\vec{M}_{Ay}\cdot\sin\left( \theta\right) \\ \vec{R}_{Ay}=\vec{M}_{Ax}\cdot\sin\left( \theta\right)+\vec{M}_{Ay}\cdot\cos\left( \theta\right) \end{aligned} \right.$ | (10) |
| --- | --- |

Finally, the final rotation coordinates of the chosen atom, *x_f_, y_f_* and **z_f_**, would be given by the following equation:

|  | $\left\{ \begin{aligned} x_{f}=\vec{R}_{Ax}+C_{\mathrm{fixed}}(x) \\ y_{f}=\vec{R}_{Ax}+C_{\mathrm{fixed}}(y) \\ z_{f}=z \end{aligned} \right.$ | (11) |
| --- | --- | --- |

After the calculation of the first atom is completed, the tool will repeat this process with all other atoms and generate a structure with rotated coordinates in a separated file.

# Perpendicular translation

The Perpendicular translation tool will shift the manipulated unit along the line that is perpendicular to the vector of mass center in the selected plane. The translation in xy-plane is shown as an example.

This tool would start with calculating the mass center of both protein units and the vector of mass centers,$\vec{M}$, can be generated for obtaining a separating vector in a similar manner to that in section 2.1.1 using the following equation:

| $\left\{ \begin{aligned} \vec{M}_{x}=C_{manipulated}\left( x \right)-C_{fixed}(x) \\ \vec{M}_{y}=C_{manipulated}\left( y \right)-C_{fixed}(y) \end{aligned} \right.$ | (12) |
| --- | --- |

| $\vert\vec{M}\vert=\sqrt{{(\vec{M}_{x})}^{2}+{(\vec{M}_{y})}^{2}}$ | | (13) | |
| --- | --- | --- | --- |
|  | $\left\{ \begin{aligned} \vec{U}_{x}=\frac{\vec{M}_{x}}{\left\vert\vec{M} \right\vert} \\ \vec{U}_{y}=\frac{\vec{M}_{y}}{\left\vert\vec{M} \right\vert} \end{aligned} \right.$ | | (14) |

| $\left\{ \begin{aligned} \vec{S}_{x}=d\cdot\vec{U}_{x} \\ \vec{S}_{y}=d\cdot\vec{U}_{y} \end{aligned} \right.$ | (15) |
| --- | --- |

Where **d** is the desired translation distance input by the user.

The separation vector $\vec{S}$ would then be rotated 90˚ or -90˚ to generate the final translation vector $\vec{T}$ using the following equation:

|  | $\left\{ \begin{aligned} \theta=\left( \pi/2 \right)\cdot\left( d/\vert d\vert\right) \\ {\vec{T}_{x}=\vec{S}}_{x}\cdot cos \left( \theta\right)-\vec{S}_{y}\cdot sin(\theta) \\ {\vec{T}_{y}=\vec{S}}_{x}\cdot sin \left( \theta\right)+\vec{S}_{y}\cdot cos(\theta) \end{aligned} \right.$ | (16) |
| --- | --- | --- |

Note that the z coordinate is not considered in this calculation as the translation is operated in the xy-plane.

Finally, this tool will modify the coordinates of the manipulated unit to create a new structure which is translated along the line perpendicular to the mass center vector by a given distance d using the following equation:

| $\left\{ \begin{aligned} x_{f}=x+\vec{T}_{x} \\ y_{f}=y+\vec{T}_{y} \\ z_{f}=z \end{aligned} \right.$ | (17) |
| --- | --- |

Similar to the rotation and spinning tool, it is also recommended to separate the manipulated unit from the fixed one to a proper distance upon using this tool in order to avoid any clashes.
